# Donor lymphocyte infusions in adolescents and young adults for control of advanced pediatric sarcoma

**DOI:** 10.18632/oncotarget.25228

**Published:** 2018-04-27

**Authors:** Sebastian J. Schober, Irene von Luettichau, Angela Wawer, Maximilian Steinhauser, Christoph Salat, Wolfgang Schwinger, Marek Ussowicz, Petar Antunovic, Luca Castagna, Hans-Jochem Kolb, Stefan E.G. Burdach, Uwe Thiel

**Affiliations:** ^1^ Department of Pediatrics and Children's Cancer Research Center, TUM School of Medicine, Technical University of Munich, Kinderklinik München Schwabing, 80804 Munich, Germany; ^2^ Medical Center for Hematology and Oncology Munich MVZ, 80639 Munich, Germany; ^3^ Department of Pediatrics, Medical University of Graz, A-8036 Graz, Austria; ^4^ Department of Pediatric Oncology, Hematology and Bone Marrow Transplantation, Wroclaw Medical University, 50-368 Wroclaw, Poland; ^5^ Department of Hematology and Regional Tumor Registry, University Hospital Linköping, 581 85 Linköping, Sweden; ^6^ Department of Oncology and Hematology, IRCCS Humanitas Cancer Center, Humanitas University, 20089, Milan, Italy; ^7^ CCC München-Comprehensive Cancer Center, DKTK German Cancer Consortium Munich, 80336 Munich, Germany

**Keywords:** donor lymphocyte infusion, allogeneic stem cell transplantation, Ewing sarcoma, rhabdomyosarcoma, alloimmunity and transplantation

## Abstract

**Background:**

Allogeneic stem cell transplantation (allo-SCT) and donor lymphocyte infusions (DLI) may induce a graft-versus-tumor effect in pediatric sarcoma patients. Here, we describe general feasibility, toxicity and efficacy of DLI after allo-SCT.

**Results:**

4 of 8 patients responded. ES#4 had stable disease (SD) for 9 months after DLI and RMS#4 partial response for 8 months with combined hyperthermia/chemotherapy. In ES#4, DLI led to SD for 6 months and reverted residual disease before allo-SCT into complete remission. After DLI, ES#4 and RMS#4 developed acute GvHD (°III–°IV), ES#4 also developed chronic GvHD. 5 patients including ES#4 lived longer than expected. Median survival after allo-SCT was 2.3 years, post-relapse survival (PRS) was 13 months. Off note, HLA-mismatched DLI were associated with a trend towards increased survival after allo-SCT and increased PRS compared to HLA-matched DLI (23 versus 3 months).

**Materials and Methods:**

We studied eight adolescents and young adults (AYAs) with advanced Ewing sarcoma (ES#1-4) and rhabdomyosarcoma (RMS#1-4) who received DLI. Escalating doses ranged from 2.5 × 10^4^ to 1 × 10^8^ CD3^+^ cells/kg body weight. AYAs were evaluated for response to DLI, graft-versus-host disease (GvHD) and survival.

**Conclusions:**

DLI after allo-SCT may control advanced pediatric sarcoma in AYAs with controllable toxicity.

## INTRODUCTION

Patients with advanced Ewing sarcoma (ES), defined as ≥ 2 bone metastases, and/or bone marrow involvement or relapse ≤ 2 years after diagnosis have poor prognoses [1]. Intensified therapy regimens including high-dose chemotherapy (HDC), involved field irradiation, autologous and allogeneic stem cell transplantation (auto- and allo-SCT) [2] could improve survival in some of these patients, excluding patients with bone marrow (BM) infiltration who do not survive irrespective of therapy [3]. Advanced rhabdomyosarcoma (RMS) patients with metastatic disease at diagnosis or those with recurrent disease have 5-year-survival rates not exceeding 30% [4].

Whereas the presence of a graft-versus-leukemia effect has been shown, a hypothesized graft-versus-tumor (GvT) effect after allo-SCT in solid pediatric tumors has not yet been proven [5–7]. In ES, an inflammatory environment may have tumorigenic and immunosuppressive effects [8]. Allogeneic stem cell transplantation (allo-SCT) may abrogate disease-maintaining homeostasis and induce a favorable antitumor immunity [9]. However, it may also cause life-threatening GvHD abolishing any beneficial effect of this approach [10]. We believe that allo-SCT has the potential to render ES and RMS susceptible to cellular immunotherapy due to the recognition of foreign histocompatibility antigens and an adjuvant effect of inflammatory responses in alloimmune reactions. Indeed, the application of tumor-redirected TCR-transgenic T cells in two ES patients with haploidentical transplants did not cause GvHD and led to a partial tumor regression in at least one patient [11]. However, due to the *Human Leukocyte Antigen* (HLA)-restriction of TCR-transgenic T cells, this treatment is currently limited to HLA-A2 positive ES patients. For all other patients, non-specific DLI may constitute an option.

To our knowledge, the role of non-specific DLI after allo-SCT in ES and RMS patients has not yet been studied systematically.

## RESULTS

### Disease outcome after DLI

**Figure 1:**
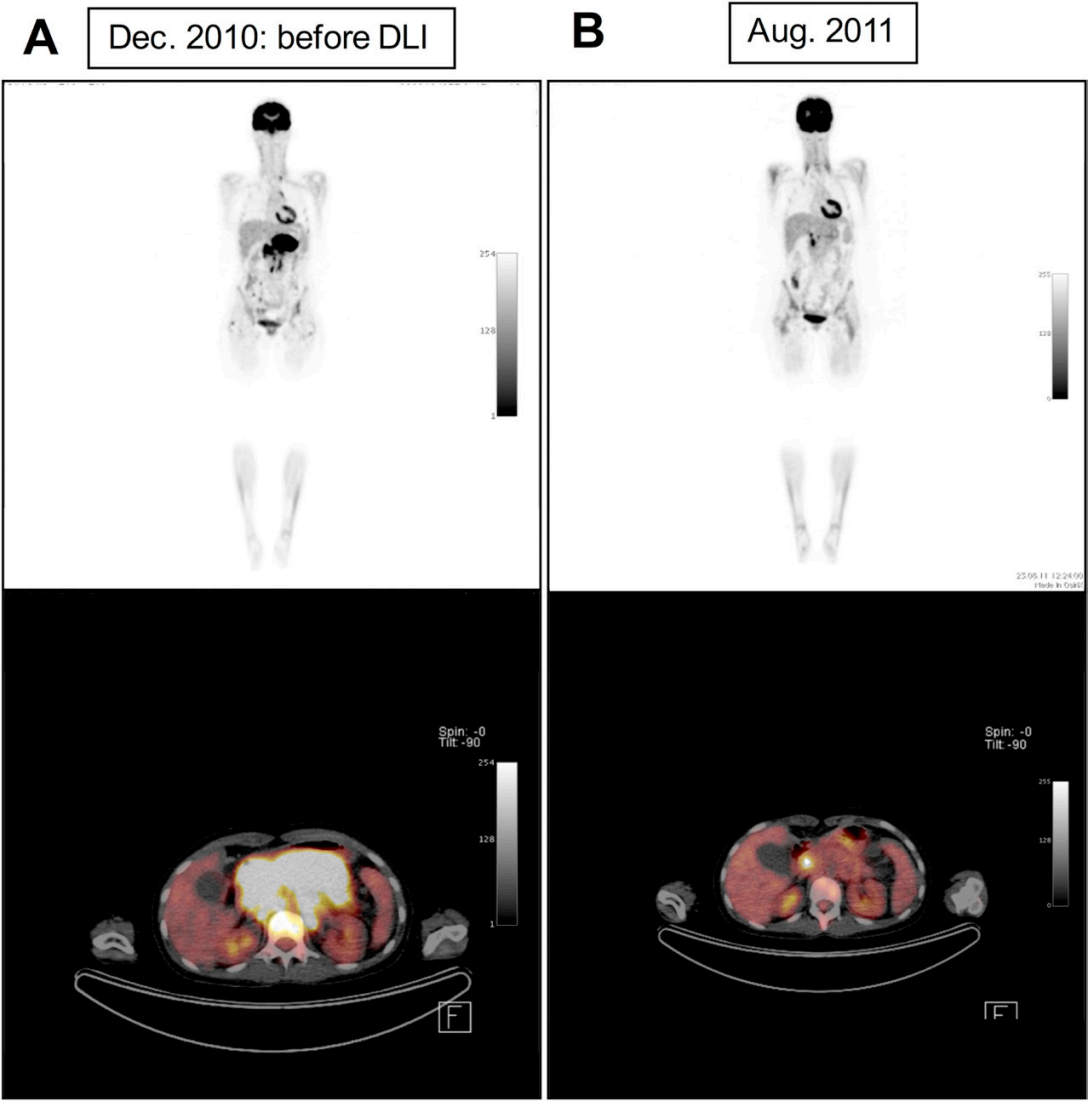
Positron emission tomography–computed tomography (PET-CT) of patient RMS#4 shows reduction of pathological [^18^F] fluorodeoxyglucose (FDG)-uptake of pancreatic tumour mass under combinatory treatment including DLI, hyperthermia and mini-ICE chemotherapy indicating partial tumor regression (**A**: before DLI, **B**: eight months after DLI).

4 out of 8 patients had a favorable clinical outcome after treatment modalities including DLI. Combination therapy of DLI and hyperthermia/chemotherapy were associated with SD for 9 months (ES#2, Supplementary Figure 1) and in patient RMS#4 with partial remission lasting 8 months (Figure 1A, 1B). Furthermore, DLI in patient ES#4 reverted residual disease before allo-SCT into complete remission and led to a course of SD lasting 6 months under repetitive DLI. Patient RMS#2 remained in CR for 97 months after his post-transplant treatment for relapsed disease including seven doses of DLI following surgical resection and salvage chemotherapy. In this analysis, responses to combination treatments including DLI were neither associated with the underlying disease nor a higher or lower frequency of certain matched or mismatched HLA-haplotypes (data not shown). Of note, 3 patients with responding clinical disease had received haploidentical grafts and DLI (ES#2, ES#4, RMS#4).

### Graft-versus-host disease after DLI

2 patients (ES#4 and RMS#4) developed acute°II–°IV GvHD after DLI and patient ES#4 suffered from extensive chronic GvHD for 108 days. Patient RMS#3 developed limited chronic GvHD after allo-SCT but not after DLI. GvHD symptoms were controlled with steroids, mycophenolat mofetil, basiliximab/etanercept, repetitive extracorporal photopheresis (ECP) and third-party mesenchymal stem cells. Altogether high doses of donor lymphocytes were relatively well tolerated and did not lead to fatal complications (Tables 1 and 2).

**Table 1:** Patient characteristics and individual outcomes

Patient Number	Gender	Age at Diagnosis	Primary at Diagnosis / Stage	Molecular Genetics	Bone marrow Involvement at Diagnosis	Reason for Allo-SCT	Auto-SCT prior to Allo-SCT	Age at Allo-SCT	Disease Stage at Allo-SCT	Hyperthermia before/after DLI for Relapse Tx	Disease Course after DLI (months)	RFS after Allo-SCT (months)	OS after Allo-SCT (months)	Post-relapse Survival (months)	Status at last Follow-up
ES#1	m	15	multifocal	EWS-FLI1 (type 1)	Yes	multifocal disease	Yes	16	CR	No	PD	13 (relapse)	24	11	DOD
ES#2	m	15	multifocal	EWS-FLI1 (type 1)	Yes	multifocal disease	Yes	16	CR	Yes	SD (7 m)	16 (relapse)	49	33	DOD
ES#3	f	17	multifocal	EWS-FLI1 (type 2)	Yes	multifocal disease	Yes	18	PD	No	PD	0 (never in CR)	5	N.A.	DOD
ES#4	f	17	local	EWS-FLI1 (type 1)	No	multifocal relapse	Yes	21	residual disease	No	CR (9 m) after 4 DLI; SD (6 m) under repetitive DLI	19 (relapse)	62	43	DOD
RMS#1	f	15	IV	UK	UK	Relapse after initial treatment	No	19	CR	No	PD	6	8	2	DOD
RMS#2	m	14	IV	embryonal	No	No CR	No	14	CR	No	CR	28	97	69	Alive in CR
RMS#3	f	25	IV	alveolar	Yes	No CR	Yes	26	CR	No	PD	10	13	3	DOD
RMS#4	f	14	IV	alveolar	Yes	No CR	Yes	14	CR	Yes	PR (8 m)	18	31	13	DOD

Abbreviations: allo-SCT = allogeneic stem cell transplantation; auto-SCT = autologous stem cell transplantation; CR = complete remission; DLI = donor lymphocyte infusion; DOD = death of disease; ES = Ewing sarcoma; GvHD = graft-versus-host disease: OS = overall survival; PD = progressive disease; PR = partial remission; RFS = relapse-free survival; SD = stable disease; UK = unknown.

**Table 2:** Graft and DLI-related information

Patient Number	Allo-SCT Conditioning	HLA match	GvHD Treatment / Prophylaxis	Onset of GvHD after allo-SCT	Acute GvHD after Allo-SCT	Chronic GvHD after Allo-SCT	Day of DLI post allo-SCT	DLI for Relapse/Progression after Allo-SCT (CD3+ per kg/BW)	Onset of GvHD after DLI	Acute GvHD after DLI	Chronic GvHD after DLI
ES#1	FLU/MEL/OKT3/TT	haploidentical (mother)	STER / CSA	d+34	Stage I-II (skin)	None	d+485, d+566, d+639, d+691, d+716	Six times; 2.5, 5, 10, 25, 100 and 500 × 10^4^	None	None	None
ES#2	FLU/MEL/OKT3/TT	haploidentical; class I matched (father)	CSA	d+63	Stage I (skin)	None	7 times; in-between d+642 and d+824	Seven times; 2.5, 5, 10, 25, 100, 500 and 1.000 × 10^4^	None	None	None
ES#3	FLU/MEL/OKT3/TT	HLA identical match (sibling)	CSA	None	None	N.A.	d+70, d+104	Twice; 10 × 10^4^ and 100 × 10^4^	None	None	None
ES#4	FLU/MEL/OKT3/TT	haploidentical (sibling)	BASX, ECP, STER / MMF, OKT3	None	None	None	N.A. after 1st four DLIs; 64 times in-between d+606 and d+917, and six times d+1081 and d+1133	74 times; 4 times to consolidate allo-SCT up to 30 × 10^4^, 64 times up to 30 × 10^4^ and six times up to 200 × 10^4^	N.A. after 1st 4 DLI, d+63 after 1st DLI / d+10 after last six DLI	twice; Stage II-III (skin) after 1st four DLIs; Stage III-IV (intestine, liver) after last six DLIs up to 200 × 10^4^	Extended
RMS#1	CRBPL/MEL/TT	HLA identical match (sibling)	UK	None	None	None	N.A.	twice; 1.000 × 10^4^	None	None	N.A.
RMS#2	BU/CTX/TT	HLA identical match (sibling)	CSA	None	None	None	N.A.	Seven times; 10, 30, 50, 100, 250, 500 and 1.000 × 10^4^	None	None	None
RMS#3	CTX/FLU	HLA identical match (sibling)	Yes, but UK	N.A.	Stage III	Limited	N.A.	once; 10.000 × 10^4^	None	None	N.A.
RMS#4	FLU/MEL/OKT3/TT	haploidentical (father)	ETN, MSC, STER / MMF, OKT3	d+49	Stage IV (intestine)	Non	d+576	once; 100 × 10^4^	d+53 (not biopsy-proven, but responsive to treatment)	Stage II-III (skin)	None

Abbreviations: allo-SCT = allogeneic stem cell transplantation; BASX = basiliximab; BU = busulfan; BW = body weight; CSA = cyclosporine A; CTX = cyclophosphamide; CRBPL = carboplatin; d = day; DLI = donor lymphocyte infusion; ECP = extracorporal photopheresis; ES = Ewing sarcoma; ETN = etanercept; FLU = fludarabin; GvHD = graft-versus-host disease; HLA = heuman leukocyte antigen; MEL = melphalan; MMF = mycophenolat mofetil; MSC = mesenchymal stem cells; N.A. = not assessable; OKT3 = anti-CD3; RMS = rhabdomyosarcoma; STER = steroids; TT = thiotepa; UK = unknown.

### Median overall survival after allo-SCT and median post-relapse survival

Altogether, 7 out of 8 patients died due to progressive disease. Median OS after allo-SCT of all patients was 27.5 months. Median OS after allo-SCT of ES patients was 36.5 months and 22 months for RMS patients. Median OS after allo-SCT for patients receiving haploidentical grafts was 40 months versus 10 months for patients receiving HLA-matched grafts. Median time to death after most recent relapse (PRS) of all patients was 13 months. Median time to death after most recent relapse for patients receiving haploidentical grafts was 23 months versus 3 months for patients receiving HLA-matched grafts.

### Individual outcomes

ES#1 was in complete remission (CR) after allo-SCT for 13 months until he suffered from a multifocal relapse. Relapsed disease was treated with irinotecan/temozolomide/temsirolimus alternating with cyclophosphamide/topotecan chemotherapy, involved field irradiation and 6 repetitive DLI with up to 5 × 10^6^ CD3^+^ cells/kg body weight. He showed no signs of GvHD after DLI. Tumor lesions did not respond to any treatment and progressed until DOD. ES#2 was in CR after allo-SCT for 16 months until a distant pulmonary metastasis was confirmed. Relapsed disease was treated with 7 DLI with up to 1 × 10^7^ CD3^+^ cells/kg. Prior to each DLI, preparative hyperthermia with concurrent ifosfamide/carboplatin/etoposide chemotherapy in reduced dosages (mini-ICE) was administered. He had stable pulmonary disease for 7 months after DLI without any signs of GvHD (Supplementary Figure 1). The patient suffered from tumor progression after 12 months (7 months after 1^st^ DLI) and received molecular targeted therapies in combination with salvage chemotherapy regimens (vorinostat/paclitaxel/vincristine), which led to a course of SD for 5 months. Afterwards, the patient became refractory to other combinatory treatments (vorinostat/sorafenib/tretinoin and rapamycin/dasatinib/temozolomide) and died of pulmonary disease progression (DOD). ES#3 had treatment-refractory PD and did not develop GvHD despite DLI (twice) with 1 × 10^6^ CD3^+^ cells/kg until she died of disease progression (DOD; Supplementary Figure 2). ES#4 had residual disease at the time of allo-SCT, which was reverted into CR after 4 DLI with up to 3 × 10^5^ CD3^+^ cells/kg. She developed extensive chronic GvHD for 108 days and stayed in CR for 19 months. Distant, localized relapse was treated with weekly DLI (60 times) with up to 3 × 10^5^ CD3^+^/kg in combination with local irradiation. This therapy regimen led to a course of SD for 6 months without any signs of GvHD. PD was again treated with 5 DLI containing up to 2 × 10^6^ CD3^+^ cells/kg in combination with weekly bevacizumab and local irradiation. Treatment was discontinued because of acute GvHD °III–°IV (gut, liver). Symptoms were controlled with steroids, mycophenolat mofetil, basiliximab and repetitive ECP. After GvHD control, disease progressed until DOD.

**Figure 2:**
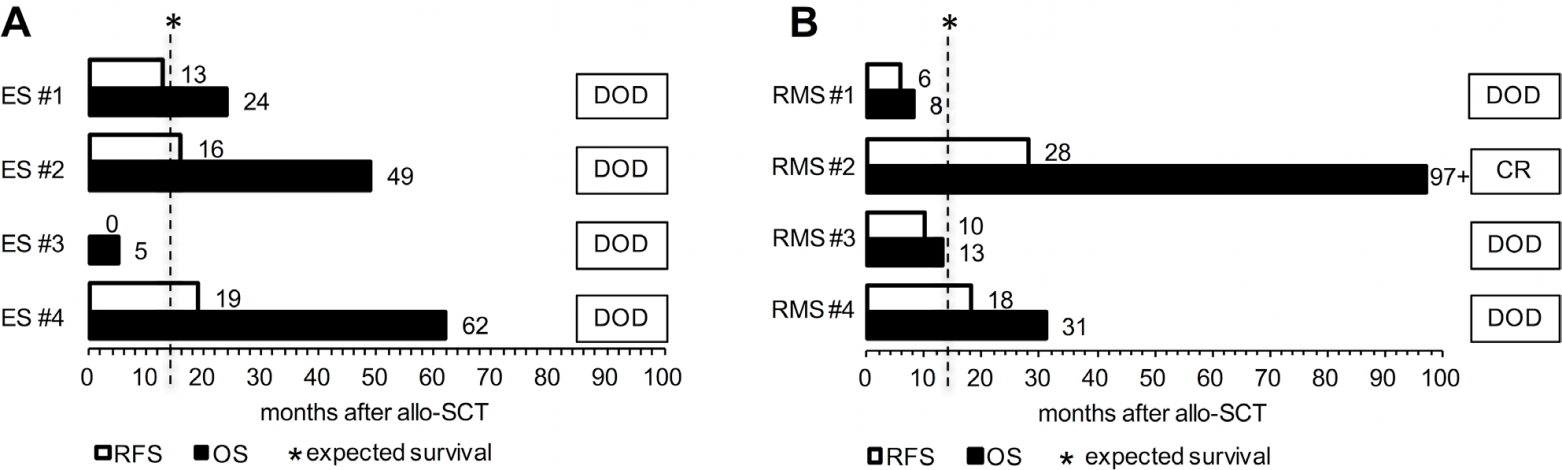
Individual survival (in months) after allogeneic stem cell transplantation is depicted for ES (**A**) and RMS patients (**B**). Abbreviations: allo-SCT, allogeneic stem cell transplantation; CR, complete remission; DOD, death of disease; ES, Ewing's sarcoma patient; OS, overall survival; RFS, relapse-free survival; RMS, rhabdomyosarcoma patient. Patients ES #1/#2/#4 and RMS #4 received HLA-mismatched, haploidentical grafts. Patients ES #3 and RMS #1/#2/#3 received HLA-matched grafts from sibling donors.

RMS#1 did not show any signs of GvHD despite 2 DLI with 1 × 10^7^ CD3^+^/kg. She relapsed 6 months after allo-SCT and died 2 months later due to disease progression. RMS#2 was in CR for 28 months after allo-SCT before he relapsed. He was then treated with surgery and chemotherapy according to the CWS 96 relapse protocol. Consecutively, 7 escalating doses of donor lymphocytes with up to 1 × 10^8^ CD3^+^/kg in combination with interleukin-2 (25 million units in total) in-between the 5^th^ and 6^th^ DLI were administered. At the time of data assessment, he was alive in CR for 97 months without any signs of GvHD. RMS#3 developed acute GvHD (°III) and limited chronic GvHD after allo-SCT. Despite break of immune tolerance, she relapsed and suffered from refractory PD after 10 months, which was treated with irradiation and one infusion of 1 × 10^8^ CD3^+^/kg donor lymphocytes. Chronic GvHD was absent before DLI. The patient then became treatment-refractory and died of disease progression (DOD). RMS#4 stayed in CR for 18 months after allo-SCT until she relapsed and received one dose of 1 × 10^6^ CD3^+^/kg donor lymphocytes upfront, as well as hyperthermia/mini-ICE. Thereafter, she developed °II–°III acute skin GvHD, which was controlled with methylprednisolone. The hyperthermia/chemotherapy regimen was then continued and the patient experienced good PR of pancreatic as well as multiple lymph node metastases (Figure 1). PR lasted for 8 months until disease progressed leading to DOD. Individual survival data for all patients is shown in Figure 2.

## DISCUSSION

Patients with advanced ES and RMS may become eligible for allo-SCT to induce a hypothesized GvT effect. This approach, however, may result in life-threatening toxicities and GvHD. In the past, reduced intensity conditioning (RIC) was implemented to reduce HDC-associated toxicity and to facilitate a GvT effect in those patients. However, reduced toxicity was bought with higher relapse rates leading to equal OS compared to HDC-based regimen and the role of allo-SCT to induce a GvT effect remained unclear [1]. Most pediatric tumors are considered poorly immunogenic compared to e.g. melanoma or lung cancer, correlating with low response rates to immune-checkpoint inhibitors [12, 13]. Further immune-evasive factors, such as low or varying HLA class I expression in-between patients and tumor sites, immune-modulatory pro-neoplastic effects of the tumor-microenvironment (TME) or high frequencies of regulatory T cells (T_regs_) have been described [14–17]. Thus, strategies to render those tumors susceptible to immunotherapeutic approaches are needed. We hypothesize that allo-SCT does not constitute an endpoint but a starting point of immunotherapy in a subgroup of patients yet to be defined. Several reports such as the use of adjuvant tumor lysate-pulsed dendritic cell vaccination in patients after standard antineoplastic treatment [18] as well as the use of natural killer (NK) cells raise hope that ES may become targetable with immunotherapy [19, 20]. In the present analysis, 4 out of 8 patients responded to treatments including DLI. Clinical responses in patient ES#2 and RMS#4 were achieved with additional hyperthermia and mini-ICE. Apart from conversion to CR (after allo-SCT) and SD with weekly DLI in patient ES#4, DLI-associated tumor responses were observed in combination approaches (e.g. hyperthermia, mini-ICE). 5 out of 8 patients lived longer than expected compared to historical study groups, including ES #4 with chronic GvHD [1, 9, 21].

Compared to survival data from non-transplanted patients with similar risk profiles [21], the patients in this study fared better. Post-relapse survival for all patients was 13 months, for ES patients 33 months and for RMS patients 8 months. Without CR2, median time to death in the study population of Ferrari et al. was 6 months [21].

Therefore, we hypothesize that there is a benefit in overall survival for the subgroup of high-risk patients in comparison to standard of care therapy that includes HDC as well. This survival benefit appears even more obvious when the subgroup of HLA-mismatched patients, who received DLI, is considered separately (40 months of median overall survival after allo-SCT for haploidentical transplanted patients versus 10 months for HLA-matched transplanted patients). In accordance, the post-relapse survival for haploidentical transplanted patients is higher than for those having received an HLA-matched graft (median time to death: 23 months versus 3 months), possibly originating from a stronger alloreactivity of donor cells against tumor alloantigens in a HLA-mismatched versus HLA-identical setting.

GvHD symptoms after DLI were absent in 6 out of 8 patients despite high doses of up to 1 × 10^8^ CD3^+^ T cells/kg. At the time of treatment, 4 DLI-non-responders had active tumor burden increasing in size, suggesting disease-triggered immune-evasive mechanisms or even a general immune-suppressive host environment.

Eliciting a pro-inflammatory environment using allo-SCT may lead to enhanced phagocytic, NK cell as well as T cell activity [22]. In this regard, there is supporting evidence that alloreactive NK cells play a role in controlling minimal residual disease (MRD) in leukemia and solid pediatric tumors and are discussed to prolong survival after haploidentical allo-SCT compared to a HLA-matched setting [19]. A recent analysis showed even lower rates of chronic GvHD rates and similar survival outcomes of haploidentical allo-SCT compared to matched sibling or unrelated donor transplants by implementing post-transplantation cyclophosphamide [23].

In the present analysis, DLI after allo-SCT are a feasible therapy option for a subgroup of advanced ES and RMS patients. Of particular importance, GvHD rates and extent were controllable and no patient died of complications. Interpretation of the present data is limited due to low patient numbers and patient heterogeneity. Nevertheless, tumor control was observed in a number of patients.

Consecutively, our data suggest that high-risk pediatric ES and RMS patients when considered for an allo-SCT, should receive haploidentical grafts and additional pre-emptive DLI, in case acute GvHD symptoms are absent.

If the allo-SCT setting has the capacity to cause immune-susceptibility in bulky tumors, there is hope to cure allo-transplanted ES and RMS patients with high-risk relapse profiles in CR using pre-emptive DLI.

## MATERIALS AND METHODS

### Data preparation and analysis

We re-evaluated data of eight patients (14–26 years) from previous publications with advanced ES and RMS, who received DLI after allo-SCT between 1997 and 2011 [3, 9]. Median follow-up after allo-SCT was 27.5 months. Inclusion criteria were diagnosis of ES or RMS (all subtypes, respectively), DLI after allo-SCT and non-participation in ongoing prospective trials. Diagnosis and relapse were confirmed by histopathology. Tumor size was monitored with CT or MR-based imaging. Disease specific chromosomal translocations were detected by molecular-genetic testing. As we describe retrospective data of a small heterogeneous group we did not perform statistical significance tests.

### Definitions

Disease was staged according to WHO classifications. Complete remission (CR) was defined as the absence of detectable disease, progressive disease (PD) as at least 25% increase in tumor volume of previous lesions despite treatment. Partial remission (PR) was defined as at least 50% tumor volume reduction. Residual disease included both PD and PR. Stable disease (SD) was defined as neither PD, PR nor CR. GvHD was graded using the Glucksberg criteria. The period of CR after allo-SCT until re-occurrence of any local or metastatic disease was termed relapse-free survival (RFS). Overall survival after allo-SCT (OS) was calculated from the time of allo-SCT until time of death. Post-relapse survival (PRS) was calculated from the time of most recent relapse after allo-SCT until death of disease (DOD). Expected median survival after allo-SCT is referred to most recent published data, i.e. 12 months for both ES [21] and RMS patients [9]. All patients and their legal guardians signed informed consent prior to treatment which was approved by the institutional review boards according to precepts established by the Helsinki Conference Declaration.

### Graft source, HLA-match and GvHD prophylaxis/treatment

7 out of 8 patients received CD3/CD19-depleted allografts from peripheral blood (PB). Only RMS#2 received a bone marrow (BM) allograft. 4 out of 8 patients (ES#3, RMS#1, #2 and #3) received HLA-matched grafts from sibling-donors. Patients ES#1, #2, #4 and RMS#4 received HLA-mismatched grafts from family members (Table 2). Conditioning regimens, GvHD prophylaxis and treatment are also listed in Table 2. Patients did not receive immunosuppressive therapy during or after DLI. When patients developed GvHD symptoms (°II), DLI were discontinued and immunosuppression was started (compare Table 2).

Information on patients’ pretreatment is elaborated in the Supplementary Materials.

## SUPPLEMENTARY MATERIALS FIGURES


