# Supplementary material for: Donor lymphocyte infusions in adolescents and young adults for control of advanced pediatric sarcoma
Source: Oncotarget. 2018 Apr 27;9(32):22741–8. doi: 10.18632/oncotarget.25228 (PMC5978262; doi:10.18632/oncotarget.25228)
Supplement: Supplementary file 1 [file oncotarget-09-22741-s001.pdf]

Donor lymphocyte infusions in adolescents and young adults for control of advanced pediatric sarcoma

SUPPLEMENTARY MATERIALS

PATIENTS' PRETREAMENT

Ewing sarcoma-patient #1 (ES #1) was diagnosed with multifocal disease and bone marrow (BM) involvement. He received chemotherapy according to Euro-Ewing 2008, proton-beam irradiation of the primary sites, tandem high-dose chemotherapy (HDC) with autologous stem cell rescue (ASCR) followed by haploidentical allogeneic stem cell transplantation (allo-SCT) in complete remission (CR). He developed acute °I-II skin graft-versus-host-disease (GvHD) for 61 days which was controlled by i.v. methylprednisolone. The patient had mixed chimerism for 13 months after allo-SCT until he suffered from a multifocal relapse, which was treated with irinotecan/temozolomide/temsirolimus and six repetitive infusions of donor lymphocytes (DLI) with up to 1×10^6 $CD3^+$ cells/kg body weight.

ES #2 received Euro-Ewing 99 protocol treatment after being diagnosed with ES metastatic to multiple bones, lungs, and BM. Due to his high-risk profile, the patient received irradiation of all tumor lesions, tandem HDC with ASCR and haploidentical allo-SCT in CR. He suffered from °I skin GvHD and therefore received immunosuppressive drugs for 120 days. After 16 months in CR, a 1st (pulmonary) relapse was treated with salvage chemotherapy and seven doses of DLI with up to 1×10^7 $CD3^+$ cells/kg in combination with hyperthermia and chemotherapy.

ES #3 was diagnosed with multifocal ES and showed disseminated BM involvement. She was treated according to Euro-Ewing 2008 protocol including chemotherapy, irradiation, consecutive tandem HDC and ASCR, followed by HLA-matched allo-SCT. Due to residual disease at the time of allo-SCT, she received two doses of DLI up to 1×10^6 $CD3^+$ cells /kg.

ES-patient #4 (ES #4) was diagnosed with localized ES and received treatment according to the Euro-Ewing 99 protocol including irradiation and HDC with ASCR. After suffering from a 1st late multifocal relapse, she received irinotecan/temozolomide based rescue therapy followed by irradiation and haploidentical allo-SCT. Due to residual disease at time of allo-SCT, DLI treatment was started with four doses of up to 3×10^5 $CD3^+$ cells/kg.

Rhabdomyosarcoma-patient #1 (RMS #1) was diagnosed with stage IV RMS and received EVAIA (etoposide/vincristine/actinomycin D/ifosfomide/adriamycin) chemotherapy regimen as well as irradiation for first line treatment. Due to relapsed disease, she became eligible for allo-SCT and was transplanted with an HLA-matched graft in CR. She relapsed six months after allo-SCT. Salvage therapy included DLI with two doses of 1×10^7 $CD3^+$/kg without any preparative treatment.

RMS-patient #2 (RMS #2) was diagnosed with disseminated embryonal RMS stage IV and showed chemo-resistant disease during first line chemotherapy according to CWS-2002 (Cooperative Soft-Tissue-Sarcoma-Study-Group). Tumor control was achieved by surgery and the patient received HLA-matched allo-SCT in CR. He relapsed 28 months after allo-SCT and received seven escalating doses of DLI up to 1×10^8 $CD3^+$/kg in combination with IL-2 once upfront, consecutive surgery and chemotherapy according to CWS 96 relapse protocol.

RMS-patient #3 (RMS #3) was diagnosed with stage IV alveolar RMS and received HDC with ASCR due to her high-risk profile. First line regimen did not induce CR, leading to eligibility for HLA matched allo-SCT. Preparative conditioning led to CR at the time of allo-SCT. Relapse occurred after 10 months and was treated with irradiation and a single dose of 1×10^8 $CD3^+$/kg donor lymphocytes.

RMS-patient #4 (RMS #4) was diagnosed with stage IV alveolar RMS and received treatment according to CWS-IV 2002 protocol, combined with radiotherapy, tandem HDC with ASCR followed by haploidentical allo-SCT in CR. She then suffered from °IV acute GvHD (intestine, skin) after allo-SCT which could be controlled by steroids, MMF, etanercept and third-party mesenchymal stem cells (MSC). In total immunosuppressive MMF was given for 10 months. Distant localized relapse occurred 18 months post-transplantation and was treated with one DLI of 1×10^6 $CD3^+$/kg in combination with hyperthermia and concurrent ifosfamide/carboplatin/etoposide chemotherapy in reduced dosage (mini-ICE).

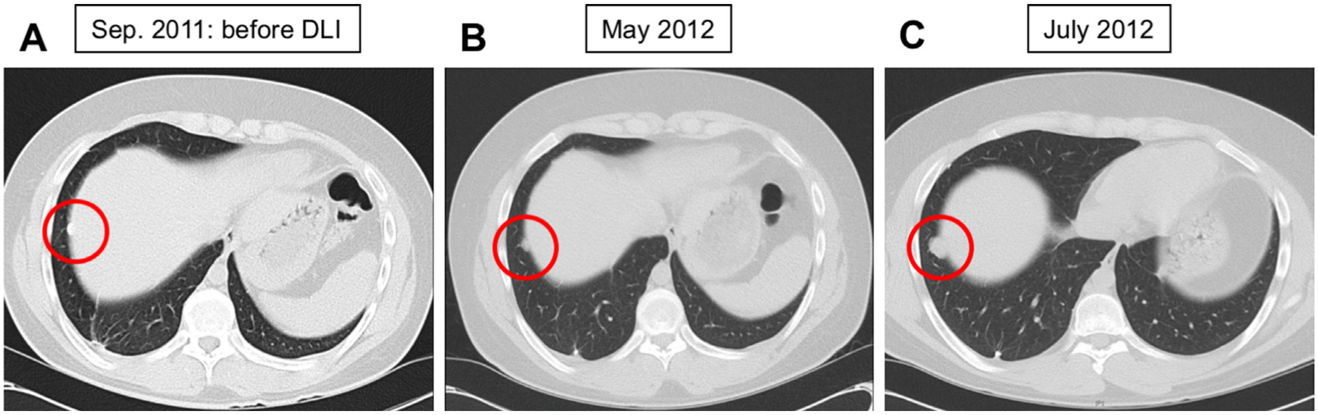

Supplementary Figure 1: Chest-CT images of patient ES#2 show stable disease lasting for seven months (compare **A** and **B**) under combinatory treatments including DLI, hyperthermia and mini-ICE (**C**: progressive disease).

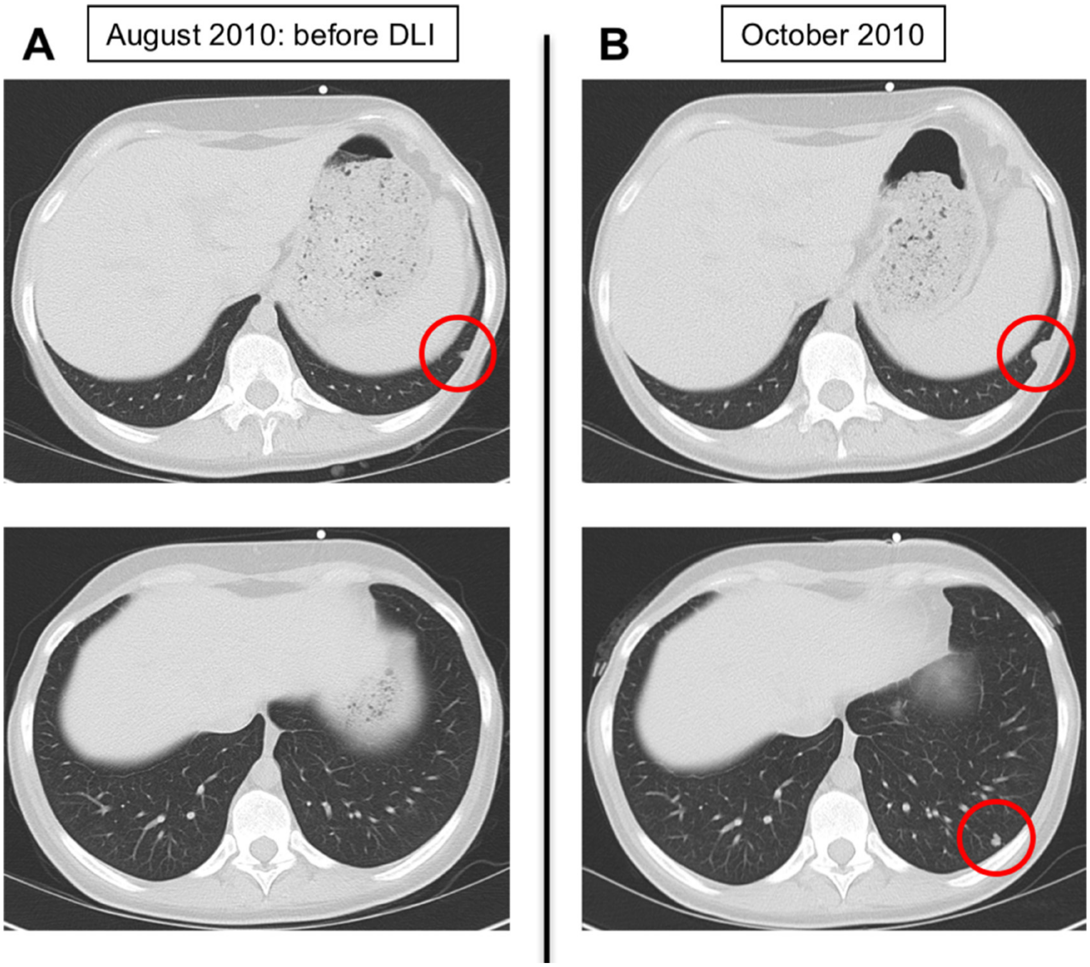

Supplementary Figure 2: Chest-CT images of patient ES#3 show progressive disease before (**A**) and after (**B**) application of two doses of 1×10^5 and 1×10^6 $CD3^+$ cells/kg body weight with the appearance of a new intrapulmonary metastasis (compare images below, red circle on the right side).
